# Simultaneous cutaneous melanoma and ipsilateral breast cancer with metastasis to the same axilla. A case report with a focus on a multidisciplinary approach

**DOI:** 10.1016/j.ijscr.2023.109119

**Published:** 2023-12-06

**Authors:** Margit Leonie Riis, Linnea Augestad, Vidar Gordon Flote, Aase Tangerud, Lars Frich

**Affiliations:** a Section of Breast and Endocrine Surgery, Department of Cancer Treatment, Oslo University Hospital, Oslo, Norway; b Locum Consultant for Melanoma, Department of Oncology, Oslo University Hospital, Norway; c Department of Oncology, Oslo University Hospital, Oslo, Norway; d Department of Radiology, Oslo University Hospital, Oslo, Norway; e Section of Oncologic Plastic Surgery, Department of Plastic and Reconstructive Surgery, Oslo University Hospital Radiumhospitalet, Oslo, Norway

**Keywords:** Breast cancer, Melanoma, Axillary metastasis, Case report

## Abstract

**Introduction and importance:**

Treatment of simultaneously occurring primary malignancies with separate lymphatic drainage is a surgical and medical challenge. We present a patient in which multidisciplinary management of coexisting melanoma and breast cancer was mandatory for optimal results.

**Case presentation:**

A 67-year-old female had a primary surgical resection for a skin lesion on the back. Histology revealed melanoma with a Breslow thickness of 4.8 mm. According to guidelines, a wide local excision was scheduled. Prior to the surgery, routine mammography revealed simultaneous ipsilateral breast cancer. A preoperative work-up revealed a pathological lymph node in the left axilla. Biopsies found metastasis from malignant melanoma. She had combined surgery with breast-conserving therapy, wide local excision of the skin on the back, and extended axillary clearance of levels I-III. Final histology revealed axillary metastases both from melanoma and breast cancer. Adjuvant therapy was decided based on a multidisciplinary approach.

**Clinical discussion:**

To our knowledge, cases of synchronous primary cutaneous melanoma with biopsy-verified axillary metastases and independent, ipsilateral primary breast carcinoma have not been described. The surgical approach was done according to guidelines. The breast cancer was re-staged based on the histology of the surgical specimen. Adjuvant treatment was a combination of treatment strategies for the two primary malignancies.

**Conclusion:**

This case highlights the need for a multidisciplinary approach in treating simultaneous breast cancer and melanoma both with axillary metastasis. The optimal treatment approach was based on close collaboration between surgeons, oncologists, radiologists, and pathologists. Multidisciplinary meetings are mandatory for optimal results.

## Highlights

• Two simultaneous primary malignancies draining to the same lymph node basin is rare.
• Post-surgical histology may cause re-staging of the primary malignancies and further direct adjuvant treatment.
• Interdisciplinary collaboration in complex oncologic care improves patient outcomes.

## 1 Introduction

We present a case with cutaneous melanoma on the left back and simultaneous ipsilateral cancer of the breast. Preoperative work-up revealed metastasis from melanoma to the left axilla. The breast tumor was initially perceived to be a stage N0 cancer.

In cases without metastases to the axilla, sentinel node diagnostics could be performed with dual tracer, with one injection of tracer at the site of the primary melanoma, and a separate injection of tracer at the site of the primary breast cancer. However, in this case, metastasis from both primaries was present in lymph nodes in the left axilla.

## 2 Presentation of case

The patient is a 67–year–old female who presented with a skin lesion on the left side of the back. An excision biopsy was performed, and histology revealed nodular melanoma with a Breslow thickness of 4.8 mm with no ulceration and 15 mitoses per mm^2^. The lesion was removed with a side margin of 5 mm and a depth margin of 8.5 mm. According to national guidelines for the treatment of melanoma, wide local excision with a 2-cm margin and sentinel node biopsy were recommended.

Prior to the wide local excision of her melanoma, screening mammography revealed a tumor in the left breast (Fig. 2a). Further diagnostics for breast cancer included an ultrasound of the breast and axilla with biopsies from both sites (Fig. 2b and c) and an equivalent MRI (Fig. 2d and e). The tumor in the left breast measured 21 × 17 mm on ultrasound. Biopsies from the breast tumor showed invasive carcinoma, NST (no special type), grade 2, estrogen and progesterone receptor-positive, and Ki67 57 %. Core needle biopsies from the tumor in the left axilla showed metastasis from melanoma. As part of the staging workup for melanoma, FDG PET/CT was performed which showed FDG uptake in the left breast and the left axilla, but no distant metastases (fig f).

After multidisciplinary discussion, a combined approach was performed with simultaneous wide local excision at the site of the primary melanoma, axillary lymph node dissection of levels I-III, and breast-conserving surgery after guidewire marking of the tumor.

**Fig. 1:**
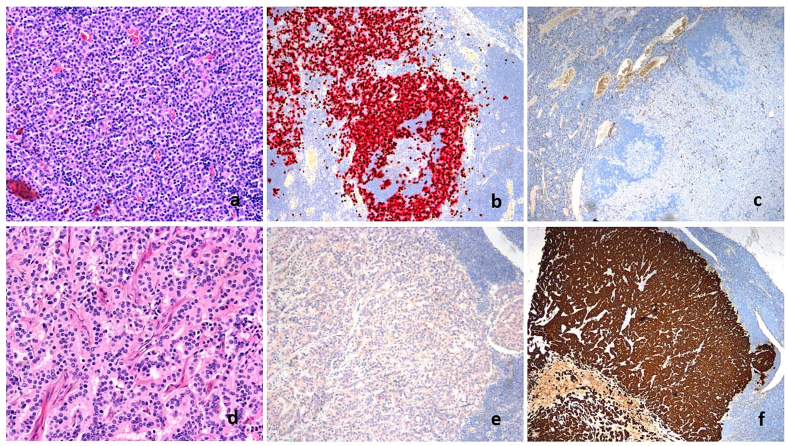
Metastases from melanoma (a-c) and breast carcinoma (d-f) in the same axillary lymph node. Diffuse, cell-rich infiltration of melanoma (a) and breast carcinoma (d) shows a trabecular arrangement with abortive gland formation in routine H&E stains. Positive staining is seen with immune markers for melanoma (b: double immune stain with SOX-10/melan-A which shows brown nuclear staining for SOX-10 and red cytoplasmic reaction for melan-A). At the same time, there is no staining seen in the same lymph node in the area with breast carcinoma cells (e). On the contrary, the AE1/AE3 (pan-cytokeratin) immunostaining shows no staining in the area with melanoma (c), while it is positive in the metastatic breast carcinoma cells (f).

Histology of the surgical specimen from the left breast revealed invasive breast carcinoma, NST, grade 3. The tumor size was 23 × 19 mm. There were clear margins. The tumor was estrogen and progesterone receptor positive and HER2 negative. Ki67 was 57 %. In the surgical specimen from the axilla, there were 14 lymph nodes, two of which had metastases from melanoma, and one with metastasis from both primary malignancies (Fig. 1). Breast cancer was re-staged to pT2N1 (1/14) M0, grade 3, stage IIb. The melanoma stage was pT4aN1b (2/14) M0, stage IIIC [1]. The metastatic lymph nodes were BRAF-positive.

**Fig. 2:**
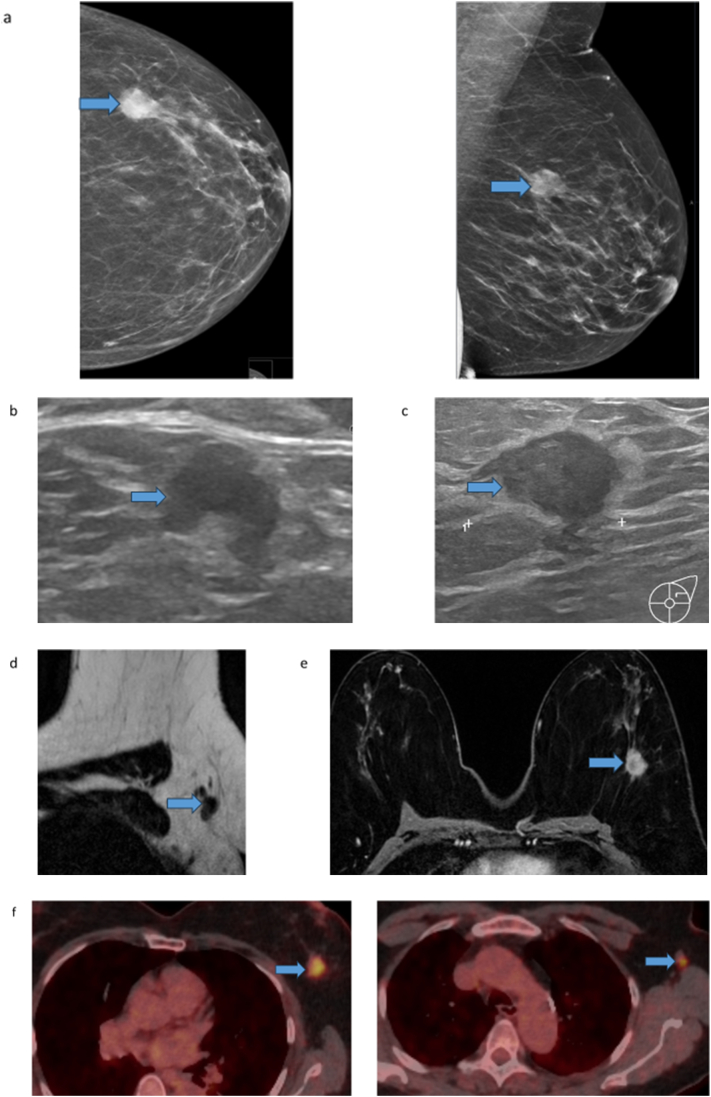
Mammography of the left breast shows a spiculated mass in the upper lateral quadrant in front and oblique view, suspicious of malignancy (blue arrow) (a). Ultrasound of the left axilla demonstrates a pathological axillary lymph node with a cortical thickness of 5 mm (blue arrow), which is highly suspicious for metastasis (b). Ultrasound of the left breast reveals a malignant irregular hypoechoic mass in the left breast (blue arrow) (c). MRI of the left axilla demonstrates a pathological lymph node with cortical thickness (blue arrow) (d). MRI of the left breast shows a spiculated malignant contrast-enhanced mass in the left breast (blue arrow) (e). PET/CT shows high FDG uptake in the tumor of the left breast and a lymph node in the ipsilateral axilla (blue arrow) (f).

The recommended chemotherapy regimen for her breast cancer was based on TNM staging [1] and the molecular profile of the tumor [2,3]. The Prosigna test revealed a molecular B-type tumor [4,5] which according to national guidelines recommends treatment with 4 cycles of anthracycline/cyclophosphamide plus weekly paclitaxel for 12 administrations, followed by endocrine therapy and zoledronic acid.

Both malignancies were high-risk tumors with a significant risk of recurrence and with strong indications for adjuvant treatment. The patient was informed about the multidisciplinary recommendations and was involved in the treatment decisions. Adjuvant combination therapy for stage III melanoma with the BRAF- and MEK-inhibitors dabrafenib and trametinib was initiated six weeks after surgery. After five weeks with BRAF- and MEK-inhibitor, 4 cycles of anthracycline/cyclophosphamide every third week were administered. Three weeks after completion of the adjuvant chemotherapy for breast cancer, adjuvant locoregional radiotherapy, 2,67 Gy × 15 towards residual breast tissue and axilla, and concomitant endocrine therapy were administered according to guidelines. Dabrafenib and trametinib were paused during chemoradiation therapy.

Multidisciplinary discussions and concern for serious adverse events due to interactions between two potentially aggressive regimens entailed a treatment pause of BRAF- and MEK-inhibitor and omission of paclitaxel. Although dabrafenib and trametinib can lead to discontinuation of therapy due to intolerance, the therapy was administered without any serious adverse advents that precluded the planned therapy. However, a single dose reduction was made due to an instance of pyrexia. The patient reported good quality of life during the treatment, with an ECOG performance status of 0–1. After 8 months of follow-up, no recurrences or new metastases occurred.

## 3 Discussion

Simultaneous primary cutaneous melanoma and ipsilateral primary breast cancer with synchronous axillary metastasis from both malignancies have to our knowledge not been described in the literature. However, a case has been reported with metastases to an axillary lymph node from breast cancer and melanoma [6]. In this case, however, the patient presented with primary breast cancer, and the metastasis from melanoma was from a melanoma excised eight years previously. Furthermore, a case has been presented with simultaneous occurrence of melanoma and breast cancer, but without metastases to the axillary lymph nodes [7]. In our case, the preoperative biopsy confirmed metastasis from melanoma to the axilla, which indicated that her melanoma was the most advanced cancer. Given the histology of the surgical specimen of the melanoma, axilla, and breast further treatment needed a multidisciplinary approach. If axillary metastasis from breast cancer had been diagnosed preoperatively without the involvement of melanoma, axillary clearance would not have been mandatory, as a clinically negative (not palpable lymph node) axilla with metastasis from breast cancer can be addressed with a sentinel node biopsy. However, if axillary metastases from breast cancer are clinically palpable, axillary clearance is advised. Thus, axillary clearance in the case of metastases from breast cancer may not be as extensive as in the case of axillary metastasis from melanoma. In the latter case, extended surgery at level I-III is recommended, as it is considered a disease with poor prognosis and without response to radiotherapy in contrast to lymph node metastases from breast cancer.

The recommended treatment for breast cancer was primary surgery followed by a combined adjuvant approach. For resectable stage III melanoma, the standard of care at the time of treatment of this patient was surgery followed by systemic therapy with either immunotherapy or BRAF- and MEK-inhibitor. However, neoadjuvant immunotherapy is emerging as the new standard of care for patients with regional metastases from melanoma [8]. Consequently, if our patient with simultaneous metastasis from melanoma and breast cancer had received neoadjuvant therapy instead of axillary clearance, the diagnosis and treatment of the breast cancer would probably have been delayed. The combination therapy of BRAF- and MEK-inhibitor dabrafenib and trametinib was approved for adjuvant treatment of resected stage III melanoma with BRAF mutation by the Food and Drug Administration (FDA) in 2018 [9], and in Norway in 2019. Dabrafenib and trametinib were preferred to immunotherapy because of their immediate response to potential microscopic disease, and the reversibility of potential toxicity upon discontinuation with a lower risk of interfering with the adjuvant treatment of the breast cancer.

This case demonstrates how the order of treatment, and the type of treatment, must be discussed at every level to optimize the prevention of cancer recurrence and minimize the risk of adverse events. The guidelines for the different malignancies [[10], [11], [12]] separately cannot be directly applied. The optimal treatment to avoid serious side effects and prevent cancer recurrence is dependent on a multidisciplinary approach and should be managed at a centralized unit [13].

This work has been reported in line with the SCARE 2020 criteria [14].

## 4 Conclusion

We present a unique clinical case of synchronous primary breast cancer and primary malignant melanoma, both with metastasis to ipsilateral axillary lymph nodes. The optimal treatment approach was based on close collaboration between plastic surgeons, breast surgeons and medical oncologists. Multidisciplinary meetings are mandatory to achieve optimal results.

## Ethical approval

Ethical approval for this case report was not required, as it solely involves the presentation of a clinical case in which the patient has given explicit written informed consent for the publication of their medical history and related data. The patient's consent, obtained in accordance with Oslo University Hospital's ethical guidelines and relevant regulatory requirements, serves as the basis for sharing this case.

## Source of funding

The authors reported no proprietary or commercial interest in any product mentioned or concept discussed in this article.

## CRediT authorship contribution statement

**Margit Riis**: Margit Riis initiated the study, was directly involved in the surgical treatment of the patient, and played a primary role in writing the manuscript.

**Lars Frich**: Lars Frich, the plastic surgeon, was actively involved in the patient's treatment and made significant contributions to the preparation, editing, and review of the manuscript.

**Linnea Augestad**: Linnea Augestad as medical oncologist specializing in melanoma, was responsible for the patient's medical treatment and made substantial contributions to editing and refining the manuscript.

**Vidar Flote**: Vidar Flote as medical oncologist specialized in breast cancer, was responsible for the patient's medical treatment and made substantial contributions to editing and refining the manuscript.

**Aase Tangerud**: Aase Tangerud, a radiologist, played a key role in editing the manuscript and was responsible for retrieving radiological images and crafting figure legends.

## Guarantor

Margit Riis is the guarantor of this study. In this role, Margit Riis takes full responsibility for the integrity and accuracy of the case report as a whole, from inception to publication. The guarantor ensures that all aspects of the study, including its design, data collection, analysis, interpretation, and the writing and review of the manuscript, meet the highest standards of academic and ethical integrity.

## Registration of research studies

N/A.

## Declaration of competing interest

None.
